# Integrated Analysis Revealed an Inflammatory Cancer-Associated Fibroblast-Based Subtypes with Promising Implications in Predicting the Prognosis and Immunotherapeutic Response of Bladder Cancer Patients

**DOI:** 10.3390/ijms232415970

**Published:** 2022-12-15

**Authors:** Hualin Chen, Wenjie Yang, Xiaoqiang Xue, Yingjie Li, Zhaoheng Jin, Zhigang Ji

**Affiliations:** Department of Urology, Peking Union Medical College Hospital, Chinese Academy of Medical Science and Peking Union Medical College, Beijing 100000, China

**Keywords:** bladder cancer, inflammatory cancer-associated fibroblast, signature, subtype, immunotherapy, prognosis

## Abstract

Inflammatory cancer-associated fibroblasts (iCAFs) are closely related to progression, anticancer therapeutic resistance, and poor prognosis of bladder cancer (BCa). However, the functional role of iCAFs in BCa has been poorly studied. In our study, two BCa scRNA-seq datasets (GSE130001 and GSE146137) were obtained and integrated by the Seurat pipeline. Based on reported markers (COL1A1 and PDGFRA), iCAFs were identified and the related signature of 278 markers was developed. Following unsupervised consensus clustering, two molecular subtypes of TCGA-BLCA were identified and characterized by distinct dysregulated cancer hallmarks, immunological tumor microenvironments, prognoses, responses to chemotherapy/immunotherapy, and stemness. Subsequently, the robustness of the signature-based clustering, in terms of prognosis and therapeutic response prediction, was validated in a GEO-meta cohort with seven independent GEO datasets of 519 BCa patients, and three immune checkpoint inhibitor (ICI)-treated cohorts. Considering the heterogeneity, re-clustering of iCAFs was performed and a subpopulation, named “LOXL2+ iCAFs”, was identified. Co-culture CM derived from LOXL2 overexpression/silencing CAFs with T24 cells revealed that overexpression of LOXL2 in CAFs promoted while silencing LOXL2 inhibited the proliferation, migration, and invasion of T24 cells through IL32. Moreover, the positive correlation between LOXL2 and CD206, an M2 macrophage polarization marker, has been observed and validated. Collectively, integrated single-cell and bulk RNA sequencing analyses revealed an iCAF-related signature that can predict prognosis and response to immunotherapy for BCa. Additionally, the hub gene LOXL2 may serve as a promising target for BCa treatment.

## 1. Introduction

Bladder cancer (BCa) is the most common urogenital malignancy in China and the sixth and tenth most incident neoplasm in the United States and worldwide, respectively [1,2]. BCa is generally categorized into non-muscle invasive bladder cancer (NMIBC) and muscle-invasive bladder cancer (MIBC). Around 70% of new cases are diagnosed as NMIBC and treated with a trans-urethral resection of the bladder tumor with/without followed intravesical instillation. Even treated with standard of care, nearly 30% of NMIBC eventually progress to MIBC. For localized MIBC, the 2-year recurrence-free survival rate, and 3-year overall survival rate decreases to approximately 50% and less than 50%, respectively [3]. BCa remains a challenge for anti-cancer management despite emerging novel treatment protocols.

Stroma is a crucial element of the tumor microenvironment (TME) and its phenotypes shift from tumor-suppressing to tumor-promoting along with the tumor development. Hijacked by cancer-derived cytokines, normal fibroblasts, a major portion of the stromal, transfer to cancer-associated fibroblasts (CAFs). Recently, mounting evidence reveals the importance of intercellular communication between cancer cells and CAFs in tumor progression and metastasis [4]. Moreover, the crosstalk induces an immunosuppressive TME via inhibiting effector T lymphocytes and enhancing inhibitory T lymphocyte function, which is responsible for impaired immunotherapeutic response [5]. Considering these challenges, a better understanding of the biological features of CAFs will increase our knowledge about the tumor-promoting and drug-resistance molecular mechanisms, and promisingly help us identify novel therapeutic targets shaping the immunotherapeutic strategies. 

The overall design of our study is illustrated in Figure S1. We integrated two BCa scRNA-seq datasets and identified a subpopulation of CAFs, the inflammatory CAFs (iCAFs). According to the Seurat protocol, we obtained the iCAF-related signature of 278 genes (Table S1). Subsequent analyses revealed the predictive abilities of the signature in prognosis and immunotherapy/chemotherapy response. Ultimately, the hub gene LOXL2 was identified and demonstrated pro-tumoral properties in various functional assays.

## 2. Results

### 2.1. scRNA-seq Analysis Highlighted the Role of iCAFs in BCa

**Figure 1:**
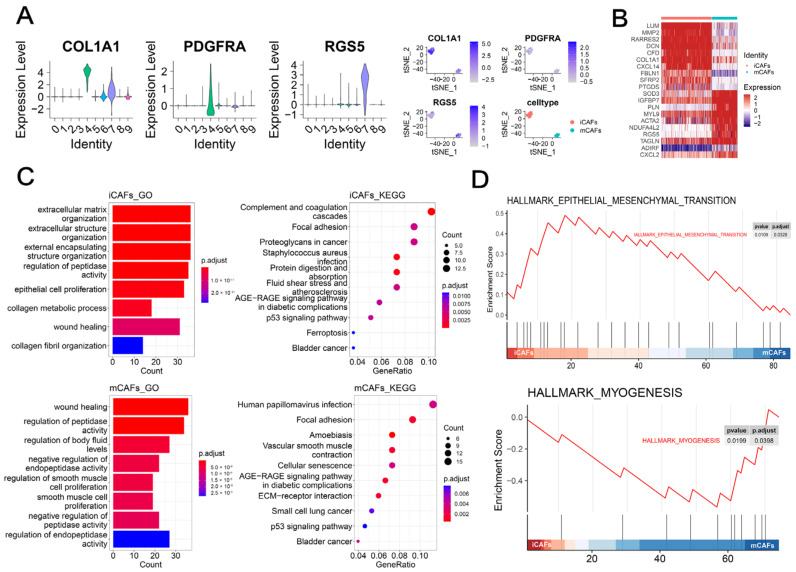
scRNA-seq analysis highlighted the role of iCAFs in BCa: (**A**) CAFs and corresponding two subpopulations (iCAFs and mCAFs) were identified by previously reported markers, including COL1A1, PDGFRA, and RGS5, respectively. Vlnplots demonstrated the expression levels of each marker in the ten clusters. t-SNE plots demonstrated the expression levels of each marker in Cluster 4 and 7 that was named iCAFs and mCAFs, respectively. (**B**) The heatmap illustrated the expression differences in the top 10 cell-type specific markers between iCAFs and mCAFs. Colors ranging from blue to red represented the expression from low to high. (**C**) GO (bar-plot, left) and KEGG (dot-plot, right) by ORA demonstrated the enriched terms by markers of iCAFs (up) and mCAFs (bottom). (**D**) GSEA showed upregulated cancer hallmarks in iCAFs (up) and mCAFs (bottom), respectively.

As illustrated in Figure 1A, ten clusters were identified with two clusters annotated as CAFs based on the marker COL1A1 [6]. Previous studies have addressed the heterogeneity and diverse properties of CAFs in various cancers [7,8]. Therefore, we further identified iCAFs (Cluster 4) and mCAFs (Cluster 7) based on previously reported markers (PDGFRA and RGS5) (Figure 1A). The heatmap showed that CXCL14, an immune and inflammatory modulator, was exclusively upregulated in iCAFs, whereas contractile proteins (TAGLN and MYL9) were overexpressed in mCAFs (Figure 1B). Enrichment analyses showed that iCAFs were related to extracellular matrix organization and epithelial cell proliferation (Figure 1C, up), whereas the focal adhesion and regulation of smooth muscle cells were mainly enriched in mCAFs (Figure 1C, bottom). Moreover, GSEA demonstrated that the epithelial–mesenchymal transition and myogenesis hallmark were upregulated in iCAFs (Figure 1D, up) and mCAFs (Figure 1D, bottom), respectively. 

Unsurprisingly, survival analysis addressed the prognostic role of iCAFs in BCa (Figure S2A). Additionally, the iCAFs subtype was related to the elder, late-stage (stage III and IV), angiolymphatic invasion, and extracapsular extension (Figure S2B).

Collectively, iCAFs, not the mCAFs subtype, were associated with progression and unfavorable prognosis of BCa patients. Therefore, the biological roles of iCAFs and cell markers were further explored in the study.

### 2.2. iCAF-Related Signature Dominated an Inflamed and Immunosuppressive TME of BCa

To shed light on the key role of iCAFs in shaping the TME, we decoded the TME contexture by several published methodologies. Firstly, based on the enrichment activities of 28 immune-related signatures, three immunity subtypes were identified and defined as high-, medium-, and low-immunity subtypes by unsupervised hierarchical clustering (Figure S3A). The high immunity subtype was related to high immune, stromal, and ESTIMATE scores and low tumor purity (Figure S3B). Moreover, high infiltration levels of CD8+ T cells and CD4+ T cells were found in the high immunity subtype. Surprisingly, both M1 and M2 macrophages were also highly infiltrated in this subtype, suggesting the heterogeneity and complexity of the TME in BCa (Figure S3C). 

Subsequently, we investigated the role of iCAFs in BCa TME by analyzing the association between iCAF-related signature and BCa immunity subtypes and found that signature was upregulated in the high immunity subtype (Figure S3A,B). Furthermore, the iCAF signature score gradually decreased from the high- to low-immunity subtype (Figure S3B). In addition to the above findings, we may speculate that iCAFs shaped an inflammatory TME of pro-tumoral phenotype in BCa. 

Previous findings unveiled that a large number of infiltrated CD8+ T cells were termed ‘exhausted’ in the TME due to intricate immunosuppressive signaling networks [9]. The exhausted subtype was characterized by overexpressed inhibitory receptors and poor effector functions. Therefore, we further explored the relevance between exhausted T cells and iCAFs and uncovered a strong and positive correlation (r = 0.77, Figure S3D). 

To verify the intriguing findings, we further deconvoluted the TME by conducting other three methodologies including quanTIseq, TIMER, and MCPcounter. Consistently, CD8+ T cells, Tregs, and M2 macrophages were highly infiltrated in the TME of BCa with high CAF-related scores (Figure S3E). 

Taken together, we speculated that iCAFs dominated a pro-tumoral inflamed and immunosuppressive TME of BCa.

### 2.3. iCAF-Related Signature Stratified BCa into Molecular Subtypes with Distinct Biological Features

**Figure 2:**
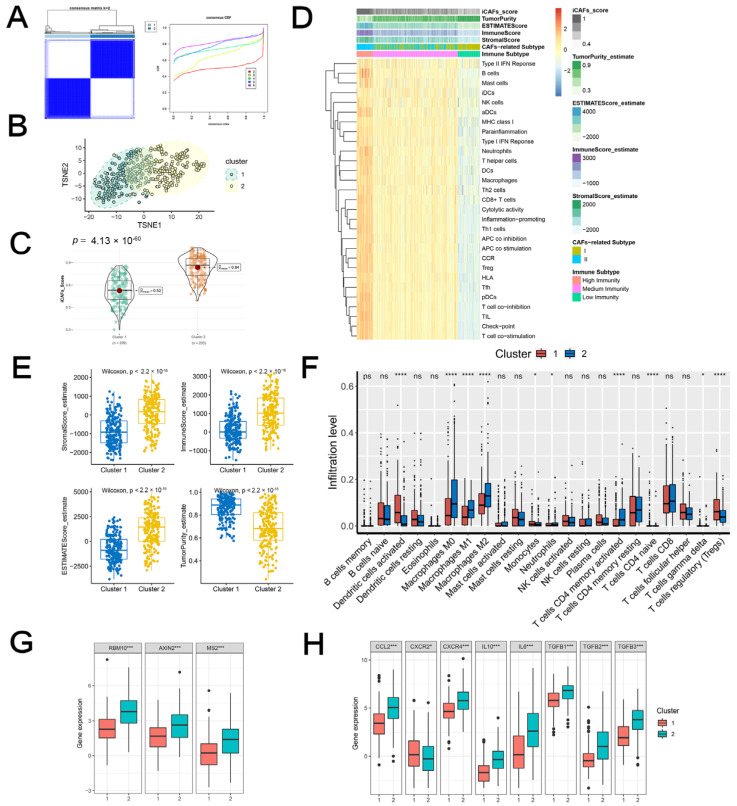
iCAF-related subtypes had distinct immunogenomic patterns: (**A**) TCGA-BLCA was classified into two molecular subtypes. (**B**) The discrimination between the two subtypes was visualized by t-SNE. (**C**) Cluster 2 was featured with a high iCAF signature score. (**D**) Heatmap demonstrated three immunity subtypes with diverse iCAF-related subtypes, ESTIMATE score, and iCAF signature score. (**E**) The distribution of stromal, immune, ESTIMATE score, tumor purity, and iCAF signature between two subtypes. (**F**) The infiltration abundances of 22 immune cells between two subtypes. The expression levels of TEX signature genes (**G**) and M2 macrophage polarization factors (**H**) between two subtypes. Ns: Non-Significant; * *p* < 0.05; *** *p* < 0.001; **** *p* < 0.0001.

Based on the iCAF-related signature, two molecular subtypes of TCGA-BLCA were identified by unsupervised consensus clustering (Figure 2A). Cluster 2 demonstrated excellent discrimination with Cluster 1, with significantly higher iCAF-related scores (Figure 2B,C).

#### 2.3.1. iCAF-related Subtypes had Distinct Immunogenomic Patterns

As illustrated in Figure 2D, a major proportion of Cluster 2 was related to the medium/high immunity subgroup, whereas Cluster 1 was characterized by the low immunity subgroup. Further deciphering the TME showed that Cluster 2 was featured by a high immune/stromal/ESTIMATE score and low tumor purity (Figure 2E). Additionally, M2 macrophages were highly infiltrated in Cluster 2, whereas dendritic cells and naïve CD4+ T cells were highly infiltrated in Cluster 1 (Figure 2F). 

Zhang et al. comprehensively analyzed the T-cell exhaustion heterogeneity in a pan-cancer manner and identified five subclusters of exhausted T cells with diverse functional properties [10]. They proposed three genes (RBM10, AXIN2, and MSI2) of the TEX signature that were highly expressed in the terminally-differentiated subtype. We analyzed the three subtype-specific makers in our molecular subtypes and found that Cluster 2 was correlated with high expression levels (Figure 2G). M2 polarization factors were also upregulated in the cluster (Figure 2H). In summary, Cluster 2 may indicate an immunosuppressive TME of BCa.

#### 2.3.2. iCAF-related Subtypes had Distinct Dysregulated Pathways

**Figure 3:**
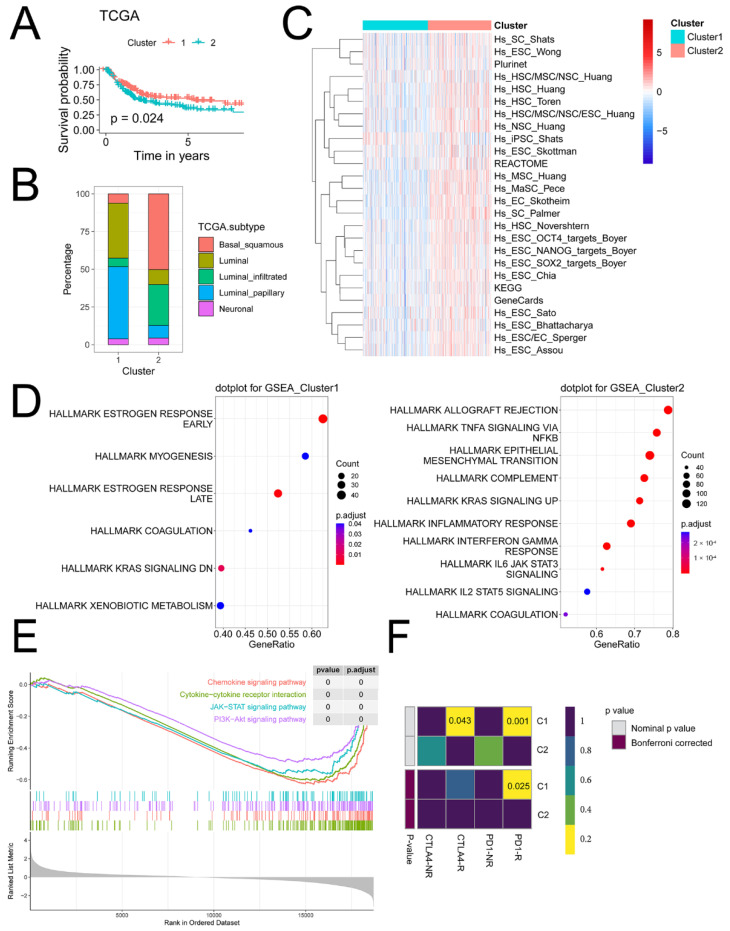
iCAF-related subtypes had distinct dysregulated pathways: (**A**) Cluster 2 was related to a worse prognosis in TCGA-BLCA. (**B**) Consensus TCGA molecular subtypes between two clusters. (**C**) The enrichment activity of 26 stemness-related signatures between two clusters. (**D**) Upregulated cancer hallmarks of Cluster 1 (left) and Cluster 2 (right). (**E**) Upregulated pathways of Cluster 2. (**F**) Immunotherapy response prediction.

Compared to Cluster 1, Cluster 2 had a poorer prognosis, a higher proportion of basal/squamous and luminal-infiltrated subtypes, and a lower proportion of luminal and luminal papillary subtypes (Figure 3A,B). Moreover, a majority of stemness-related signatures were upregulated in Cluster 2 (Figure 3C).

Dysregulated hallmarks of cancer were then investigated in molecular subtypes. Results revealed that Cluster 2 was related to upregulated EMT, the TNF-α/NF-κB pathway, and IL-6/JAK/STAT3 signaling (Figure 3D). GSEA revealed that Cluster 2 was related to the chemokine signaling pathway, cytokine–cytokine receptor interaction, JAK/STAT, and PI3K/AKT signaling pathway (Figure 3E).

#### 2.3.3. iCAF-related Subtypes Hold Diverse Responses to Immunotherapy and Chemotherapy

As predicted by GenePattern, we found that Cluster 2 was less sensitive to anti-PD1 therapy (Figure 3F).

Moreover, Cluster 2 also held lower sensitivity to six chemotherapeutic agents including cisplatin, doxorubicin, gemcitabine, methotrexate, paclitaxel, and vinblastine, which were commonly used in urological practices (Figure S4).

### 2.4. iCAF-related Subtypes Demonstrated Robustness in the External GEO-meta Dataset

To validate these findings, seven independent BCa datasets of 519 patients with clinical information from the GEO database were normalized into a meta-cohort (Figure S5A). 

Two clusters were identified (Figure S5B) and Cluster 1 was mainly divided into medium/high immunity groups (Figure S5C). A positive correlation between iCAFs and TEX verified the immunosuppressive role of iCAFs in the inflamed TME (r = 0.7, Figure S5D). Additionally, M2 polarization factors were overexpressed in Cluster 1 (Figure S5E). 

Consistent with previous findings, the iCAF-related subtypes predicted BCa survival and molecular subtypes (Figure S5F,G). Pathway analysis unveiled that the upregulated genes of Cluster 1 mainly participated in cytokine regulation, inflammation, and immunomodulation (Figure S5H). 

Ultimately, the response to therapy prediction performance of the iCAF-related subtypes was evaluated. Results showed that Cluster 2 was characterized by more sensitivity to anti-PD1 therapy and chemotherapy (Figure S5I,J).

### 2.5. iCAF-related Subtypes Predicted Prognosis and Immunotherapeutic Response in ICI-Treated Cohorts

**Figure 4:**
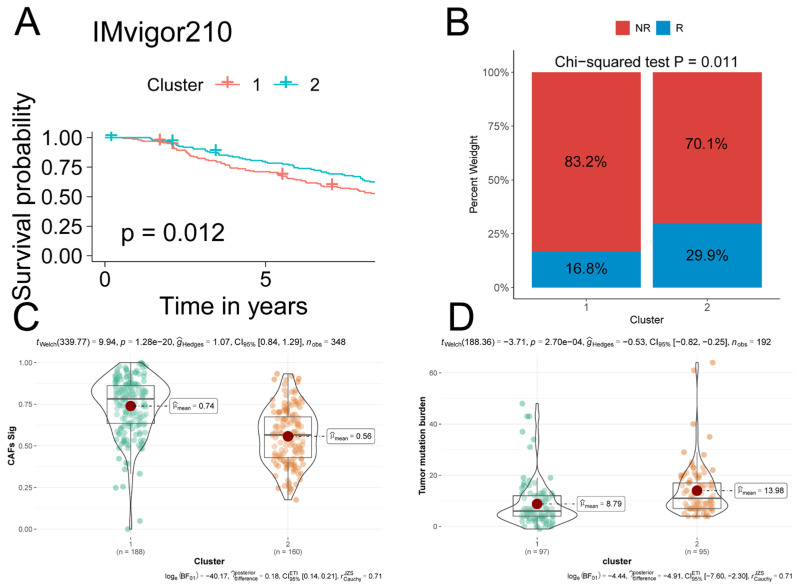
iCAF-related subtypes predicted prognosis and immunotherapeutic response in IMvigor210: (**A**) Two clusters were identified through iCAF-related signature-based consensus clustering. Patients in Cluster 1 were related to a poorer prognosis compared to those in Cluster 2. (**B**) A stacked bar-plot showed that Cluster 1 had a higher proportion of responders compared to Cluster 2. *p* value was determined by Chi-squared test. R: responder, NR: non-responder. (**C**) Cluster 1 had significantly higher iCAF-related scores compared to Cluster 2. iCAF-related score of each IMvigor210 sample was determined by ssGSEA. (**D**) Cluster 1 was related to high TMB.

Via unsupervised consensus clustering, the IMvigor210 cohort was divided into two molecular subtypes based on the iCAF-related signature. Cluster 1 with a higher iCAF-related signature score predicted a poorer prognosis and lower immunotherapy response rate compared to Cluster 2 (Figure 4A–C). Moreover, tumor mutation burden (TMB), a predictor of immunotherapeutic response, was significantly lower in Cluster 1 (Figure 4D).

Although no significance was observed in GSE135222 and GSE78220, the cluster with a high iCAF-related signature score was related to a relatively poor prognosis and impaired immunotherapeutic response (Figure S6).

### 2.6. LOXL2+ iCAFs Predicted Poor Prognosis

**Figure 5:**
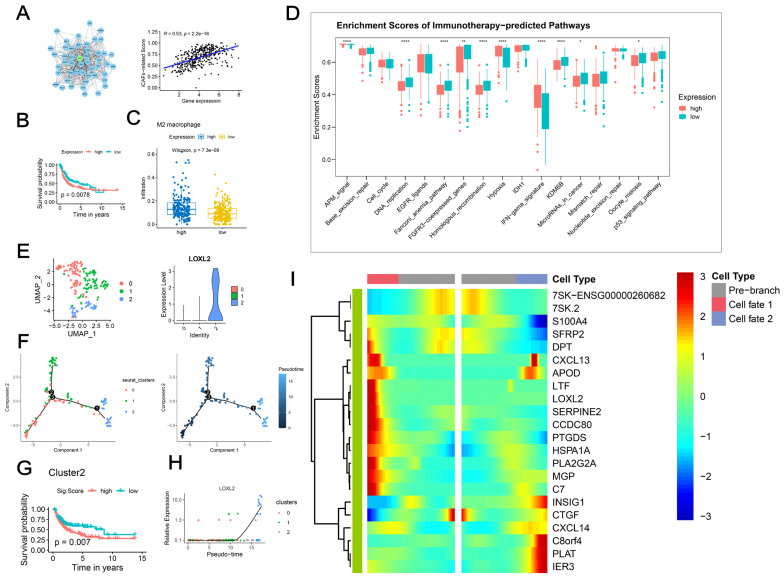
LOXL2+ iCAFs predicted poor prognosis: (**A**) Hub gene LOXL2 was identified by analyzing the PPI network. (**B**) High expression of LOXL2 was associated with poor prognosis. (**C**) The infiltration level of M2 macrophage was significantly higher in the overexpressed LOXL2 group. (**D**) Highly expressed LOXL2 was related to several downregulated immunotherapy-predicted pathways. * *p* < 0.05; ** *p* < 0.01; **** *p* < 0.0001. (**E**) Re-clustering of iCAFs identified three subpopulations and LOXL2 was highly expressed in subcluster 2. (**F**) Pseudotime analysis demonstrated that subcluster 0 was projected onto the root while subcluster 1 and 2 were projected onto two branches, respectively. (**G**) Subcluster 2 was related to a worse prognosis. (**H**) LOXL2 expression level varied dramatically with the trajectory tree. (**I**) Heatmap demonstrated the branch-dependent genes.

Through PPI network analysis, LOXL2 was identified as the hub gene of the iCAF-related signature (Figure 5A). Pearson correlation analysis suggested a positive significant correlation between LOXL2 and the iCAF-related score (r = 0.53, Figure 5A). Additionally, the overexpression of LOXL2 predicted an unfavorable prognosis (Figure 5B). The immunological analysis demonstrated that LOXL2 overexpression was associated with a high infiltration level of M2 macrophage (Figure 5C). Moreover, highly expressed LOXL2 suggested low enrichment scores of several immunotherapy-predicted pathways (Figure 5D). 

Considering the heterogeneity of iCAFs, we re-clustered iCAFs and successfully identified three subpopulations. LOXL2 was exclusively overexpressed in Cluster 2, which was associated with poor prognosis (Figure 5E,G). Pseudotime analysis demonstrated that subcluster 0 was projected onto the root while subcluster 1 and 2 were projected onto two branches, respectively (Figure 5F), and LOXL2 expression level varied dramatically with the trajectory tree (Figure 5H). Interestingly, we found that branch point 1 functioned as a “checkpoint” regulating the differentiation and gene expression profiles. Therefore, we performed BEAM analysis to identify branch-dependent genes. Results demonstrated that modules including CXCL13, LOXL2, C7, and APOD were positively correlated with the developmental direction of subcluster 2 of iCAFs (Figure 5I). Hence, we named this cluster “LOXL2+ iCAFs”.

### 2.7. Upregulated LOXL2 in CAFs Promoted the Proliferation, Migration, and Metastasis of BCa Cells

**Figure 6:**
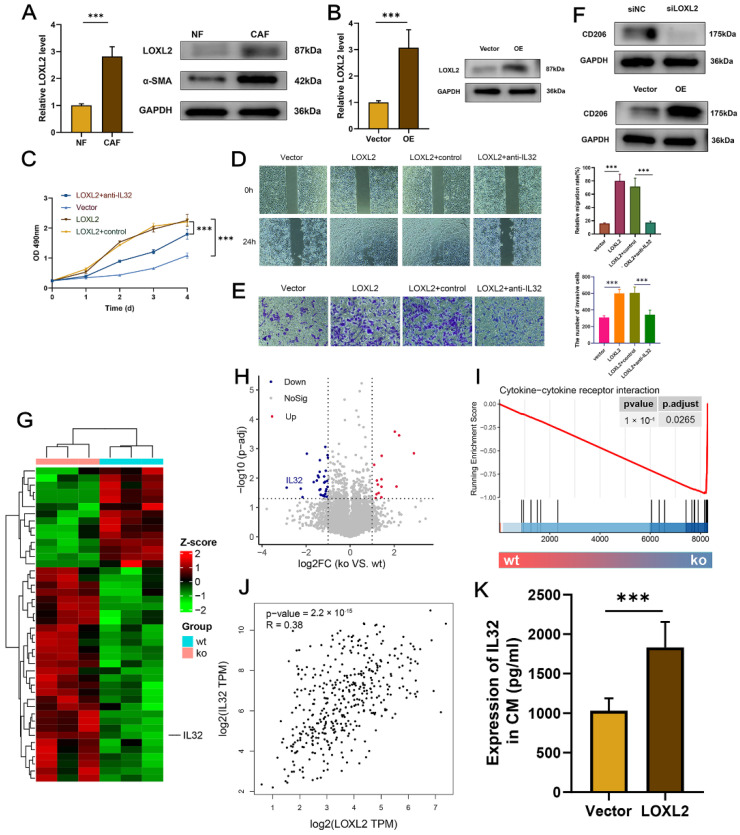
Upregulated LOXL2 in CAFs promoted the proliferation, migration, and metastasis of BCa cells: (**A**) LOXL2 and α-SMA were upregulated in CAFs at mRNA and protein levels. (**B**) qRT-PCR and WB verified the efficacy of overexpression. (**C**) CCK-8 assay demonstrated the pro-proliferation of LOXL2 in CAFs together with or without anti-IL32. (**D**,**E**) T24 cells were treated the same as in (**C**), and the wound-healing assay (**D**) and Transwell assay (**E**) were carried out to evaluate the migrative and invasive potentials of tumor cells. (**F**) CD206 protein level was affected by conditioned LOXL2. (**G**,**H**) IL32 was downregulated in the siLOXL2 group. Heatmap demonstrated the top DEGs of each group. The threshold for volcano plot was |log2FC| >1 and adj.*p*.Val. < 0.05. (**I**) Enriched term in siLOXL2 group. (**J**) Correlation between LOXL2 and IL32 (GEPIA, http://gepia.cancer-pku.cn/index.html, accessed on 9 December 2022). (**K**) ELISA demonstrated the IL32 protein expressions in the culture supernatant of CAFs with conditioned LOXL2. *** *p* < 0.001.

We found that LOXL2 was endogenously upregulated in CAFs, compared to NFs (Figure 6A). To investigate the biological roles of LOXL2, gain and loss of function were performed by plasmid and siRNA, respectively, and the efficacy was verified by qRT-PCR and WB assays (Figure 6B and Figure S7A).

The co-culture system revealed that the overexpression of LOXL2 in CAFs promoted, while the silencing of LOXL2 inhibited, the proliferation (Figure 6C and Figure S7B), migration (Figure 6D and Figure S7C), and invasion (Figure 6E and Figure S7D) of T24 cells. 

Considering the immunosuppressive TME dominated by LOXL2+ iCAFs, we evaluated the expression of the M2 polarization marker CD206 in CAFs-CM with conditioned LOXL2. Immunoblotting assays showed that the depletion of LOXL2 significantly diminished the CD206 protein level (Figure 6F, up). Contrarily, enhanced LOXL2 expression increased the protein level (Figure 6F, bottom). 

To figure out the mechanisms that CM derived from LOXL2 overexpression/silencing fibroblasts regulated T24 cells, RNA-seq was processed after LOXL2 knockdown in CAFs (Figure 6G). IL32 was identified to be the only soluble factor among the top DEGs (Figure 6G,H). GSEA showed that cytokine–cytokine receptor interaction was enriched in DEGs of LOXL2 knockdown (Figure 6I). Moreover, the online tool GEPIA showed a positive correlation between LOXL2 and IL32 (r = 0.38, Figure 6J). ELISA showed that IL32 was positively regulated by LOXL2 (Figure 6K and Figure S7E). Phenotypic assays were then performed to investigate the functional relationship between LOXL2 and IL32. The CCK-8 assay revealed anti-IL32 inhibited BCa cell proliferation that was induced by CM derived from LOXL2 overexpression CAFs (Figure 6C). Similar trends were found in wound healing (Figure 6D) and transwell invasion assays (Figure 6E). In the co-culture system with CM derived from LOXL2 silencing CAFs, rescue assays showed that rIL32 could restore the BCa cell’s proliferative, migration, and invasive properties inhibited by LOXL2 knockdown (Figure S7B–D). 

Collectively, CAFs may promote BCa cell development through the LOXL2-IL32 axis.

## 3. Discussion

A growing number of studies have suggested the roles of CAFs in tumor progression, metastasis, and drug resistance [11,12]. CAFs serve two main roles in TME: either pro-tumorigenic or tumor-inhibiting function. Considering the heterogenicity of CAFs, subtypes identification can provide promising targets for pro-tumorigenic CAFs. Öhlund and colleagues uncovered the heterogenicity of CAFs with TME of pancreatic cancer and identified two subtypes, iCAFs and mCAFs, with distinct biological properties [8]. Subsequently, Chen et al. performed a scRNA-seq analysis on BCa and identified these two subtypes of CAFs [7]. As demonstrated in their study, iCAFs had critical roles in tumor progression and indicated poor prognosis. Therefore, our study was designed and analyzed based on this evidence. 

In the study, we constructed an iCAF-related signature and stratified BCa into two molecular subtypes with diverse functional heterogenicities including immune cell infiltration, dysregulated pathways, prognosis, and response to immunotherapy/chemotherapy. The upregulation of the hub gene LOXL2 of the signature was associated with M2 macrophage infiltration and resistance to immunotherapy. Moreover, subcluster 2 identified from iCAFs re-clustering showed exclusive overexpression of LOXL2 and suggested poor prognosis. 

Enrichment analysis of the signature revealed that iCAFs were related to ECM organization, cell proliferation, wound healing, focal adhesion, and EMT. Moreover, TCGA-BLCA Cluster 2 with upregulated signature was related to EMT, IL-6/JAK/STAT3 signaling, and the TNF-α/NF-κB pathway. In combination with crosstalk with tumor cells (chemokine signaling pathway and cytokine–cytokine receptor interaction), iCAFs were responsible for tumor initiation, progression, invasion, and metastasis, consistent with previously published studies [7]. 

Unsupervised clustering identified two iCAF-related subtypes with distinct molecular features in both TCGA-BLCA and GEO-meta datasets. Clusters with an upregulated signature were characterized by inflamed TME, unfavorable prognosis, low response rate to immunotherapy/chemotherapy, high infiltration of M2 macrophages, and dysregulated cancer hallmarks. Although a large number of studies stated that the inflamed TME induced by CD8+ T cells suggested anti-tumor functions and favorable prognosis, our findings showed that iCAFs may also induce the inflamed TME with pro-tumoral properties, possibly due to secreted pro-inflammatory cytokines and factors [13]. CD8+ T cells infiltrated into the TIME became “exhausted” at a large proportion. The exhausted T cells were characterized by elevated inhibitory markers and poor effector function. The exhausted T cells can be mainly classified into two subsets: progenitors with highly expressed elevated inhibitory markers and a high response rate to immunotherapy, and the terminally differentiated with a negative impact on survival. In the study, a significantly positive correlation was addressed between the TEX-associated signature and iCAF-related signature. Furthermore, three terminally differentiated subtype-specific genes including RBM10, AXIN2, and MSI2 were positively correlated with the iCAF-related signature and overexpressed in the cluster of the high iCAF-related signature score (Cluster 2 of TCGA-BLCA). Infiltrated M2 macrophages with upregulated M2 polarization factors and high abundances of Tregs also supported these findings. Their presence in TME shaped an immunosuppressive phenotype and, correspondingly, the response to immunotherapy was impaired in this cluster. In combination with results from the validated cohorts (GEO-meta and ICI-treated cohorts), the signature demonstrated satisfactory performance in predicting prognosis and immunotherapeutic response. 

In our study, clusters with high iCAF-related signature scores demonstrated less sensitivity to chemotherapeutic agents. As the most critical stromal cells in TME, CAFs participated in ECM remodeling and induced the stiffness feature of ECM. As a result, the chemotherapeutic agent can be hardly delivered to the tumor core, resulting in the resistance of BCa to chemotherapeutic drugs. Moreover, a positive association between iCAFs and stemness was addressed in our study, suggesting the potential role of iCAFs in cancer stemness modulation. Reciprocally, cancer stemness challenged the effective treatment by shaping an immunosuppressive TME. Therefore, it is reasonable that targeting the iCAFs and destructing the stiffness may be appealing strategies to enhance the treatment efficacy. 

In our study, CAFs with overexpressed LOXL2 facilitated the proliferation, migration, and invasion of BCa cells via IL32. Targeting LOXL2 in decreasing tumor cell growth and metastasis has been well-documented in various tumors such as gastric cancer [14], pancreatic cancer [15], and cervical cancer [16]. Consistent with our study, the negative effects of upregulated LOXL2 in immunotherapy have also been addressed in lung cancer by Pent et al. [17]. In their study, LOXL2 suppression eliminated exhausted T cells and abrogated resistance to PD-L1 blockade. Moreover, Gong et al. found that LOXL2 enhanced resistance to chemotherapy drugs by activating the ITGA5/FAK/PI3K/ROCK1 signaling pathway in liver cancer, whereas silencing LOXL2 restored the efficacy of the drug [18]. Taken together, inhibition of LOXL2 may be a promising strategy in CAF-targeting management, in terms of inhibiting tumor progression and restoring therapeutic efficacy.

## 4. Materials and Methods

### 4.1. Data Acquisition

Two bladder cancer scRNA-seq datasets were downloaded from the GEO database by accession numbers: GSE130001 and GSE146137 (mice data were discarded) [19,20]. BCa bulk RNA-seq datasets with survival data were procured from the GDC portal of TCGA and GEO databases: TCGA-BLCA, GSE5287, GSE13507, GSE31684, GSE48075, GSE48277, GSE69795, and GSE70691. The seven GEO datasets of 519 patients were integrated and batch effects were adjusted by Combat [21]. Three PD-(L)1 treated datasets with clinical traits were also downloaded: IMvigor210 (BCa) [22], GSE78220 (melanoma) [23], and GSE135222 (lung cancer) [24]. Then, 26 stemness-related gene sets were downloaded from a web-based tool: StemChecker (http://stemchecker.sysbiolab.eu/, accessed on 12 November 2022) [25]. The T-cell exhaustion (TEX)-associated signature and 28 immune-related signatures were obtained from the supplementary files of the study by Zhang et al. [10] and Wang et al. [26], respectively.

### 4.2. iCAF-related Signature Identification

We performed the quality control progress as previously described [27]. The normalization, integration, dimension reduction, and clustering were conducted stepwise according to the Seurat manual [28]. Based on previously reported markers, we first identified CAFs from the integrated scRNA-seq datasets [7]. Re-clustering was further performed in CAFs to identify iCAFs and myo-cancer-associated fibroblasts (mCAFs) subpopulations. 

We then used the FindMarkers function with logfc.threshold = 1, to identify markers of iCAFs that formed the iCAF-related signature.

### 4.3. Enrichment Analyses

The enrichment of iCAF-related signature and relative activities of other signatures/pathways were quantified by single-sample Gene Set Enrichment Analysis (ssGSEA) of the GSVA package [29]. 

Over Representation Analysis (ORA) was performed to determine the enriched terms of biological functions or processes in iCAFs/mCAFs-related signatures. Gene Set Enrichment Analysis (GSEA) was performed to evaluate the biological features between molecular subtypes. R packages clusterProfiler was utilized to perform ORA and GSEA and visualize the results [30]. 

### 4.4. Unsupervised Clustering and Biological Properties Exploration

Based on the iCAF-related signature, we performed unsupervised clustering using the R package ConsensusClusterPlus [31]. The performance of clustering was visualized by t-SNE [32]. Hierarchical clustering based on the enrichment activities of 28 immune-related signatures was performed to identify immune-related subtypes.

Various TME decoding methodologies were used to determine the composition of the tumor immune microenvironment (TIME), including ESTIMATE [33], CIBERSORT [34], quanTIseq [35], TIMER [36], MCPcounter [37]. Analysis was performed via R packages IOBR [38].

The proportion of consensus molecular subtypes based on TCGA [39] and UNC [40] classifications was evaluated by the BLCAsubtyping R package [41]. 

Response to chemotherapy and immunotherapy was evaluated by the pRRophetic R package [42] and a web platform GenePattern [43], respectively. 

### 4.5. Hug Gene Identification 

Genes of iCAF-related signature were subjected to the STRING database (https://cn.string-db.org/, accessed on 16 November 2022) for protein–protein interaction (PPI) networks functional enrichment analysis. The generated network was analyzed by a plugin named cytoHubba of Cytoscape to identify the hub gene. To be specific, there were 11 methods implemented in the plugin for hub gene identification. We applied each of the 11 methods to the PPI network and obtain the hug gene by comprehensively analyzing the results. 

### 4.6. Cell Lines, Culture, Transfection, and Reagents

We obtained human bladder cancer cell line T24 from the Cancer Institute of the Chinese Academy of Medical Sciences. The cell lines were cultured in Dulbecco’s modified Eagle’s medium (DMEM), supplemented with 10% fetal bovine serum (FBS) and 1% penicillin/streptomycin (Gibco, Grand Island, NY, USA). The cell line was grown at 37 °C in a humidified atmosphere of 95% air and 5% CO_2._

Primary fibroblasts were isolated as described in a previous study [44]. In brief, tissues from tumor and non-tumor were firstly aseptically excised and minced with scissors and forceps. After digested in type I collagenase (Gibco, Grand Island, NY, USA), cells were cultured at 37 °C with the culture medium of DMEM with 10% of FBS. The primary fibroblasts were used in the 3–12 passage, mainly in the 6 passage.

We purchased pcDNA3.1/LOXL2 (negative control: pcDNA3.1) and small interfering RNA (siRNA) targeting LOXL2 (si-LOXL2) (negative control: si-NC) from RiboBio (Guangzhou, China). Following the manufacturer’s guidelines, cells were transfected using Lipofectamine 3000 (Invitrogen, Carlsbad, CA, USA).

Human recombined IL32 (rIL32) was purchased from R&D Systems (Minneapolis, MN, USA). 

### 4.7. Quantitative Real-Time Reverse Transcription-Polymerase Chain Reaction (qRT-PCR)

Total RNA samples from cell lines were first extracted with TRIzol (Life Technologies, Gaithersburg, MD, USA), and SYBR Premix Ex Taq II kit (TAKARA, Tokyo, Japan) was used for the qRT-PCR assay. The target gene mRNA expression was quantitated and normalized to GAPDH. The primers of LOXL2 were 5′-GGGTGGAGGTGTACTATGATGG-3′ (forward) and 5′-CTTGCCGTAGGAGGAGCTG-3′ (reverse). The primers of GAPDH were 5′-GGAGCGAGATCCCTCCAAAAT-3′ (forward) and 5′-GGCTGTTGTCATACTTCTCATGG-3′ (reverse). All the experiments were replicated three times. 

### 4.8. Western Blotting and Antibodies

The cells were lysed in RIPA lysis buffer. Protein concentration was measured by a BCA assay kit (Beyotime, Shanghai, China). Protein lysates were separated using 10% SDS-PAGE and transferred onto PVDF membranes. The membranes were blocked with 5% skimmed milk for 1 h at room temperature (RT) and then incubated with primary antibody overnight at 4 °C. Following this, the membranes were incubated with the secondary antibody at RT for 1 h. Each blot was detected by an ECL kit. 

Primary antibodies used: anti-LOXL2, anti-αSMA, anti-CD206, anti-IL32, and anti-GAPDH. All antibodies were purchased from Sigma-Aldrich (St. Louis, MO, USA). 

### 4.9. Conditioned Medium (CM) Derived from Fibroblasts

CM from CAFs with conditioned LOXL2 or NFs was obtained as previously described [45]. In brief, fibroblasts were first cultured in DMEM without FBS after being washed with PBS. CM was then collected and the supernatant was stored at −20 °C for the following experiments. 

### 4.10. Cell Proliferation Assay

The proliferation assay was performed according to the manufacturer’s protocols (Beyotime, Shanghai, China) as previously described [46]. In brief, Cell Counting Kit 8 (CCK-8, Abcam, Cambridge, UK) was used to perform the cell proliferation assay. T24 cells (1 × 10^4^) were cultured in 96-well plates with CM from CAFs with conditioned LOXL2. After 48 h culture, the medium was replaced with CCK-8 solution and 4 h incubation was followed at 37 °C. The absorbance was measured at 450 nm. 

### 4.11. Wound-Healing and Transwell Assays

T24 cells were cultured until confluency and a wound was created using a 200 µL pipette tip. Then, cells were incubated were cultured in the medium of DMEM with 2% FBS and 50% CM derived from CAFs with conditioned LOXL2. The wound areas were photographed every 3 h and cell migration was analyzed after 24 h. 

The two-chamber Matrigel invasion assay was used to perform the transwell assay. T24 cells were seeded in the upper chamber and 500μL CM from CAFs with conditioned LOXL2 was added to the lower chamber. After culturing for 72 h, cancer cells under the filter were stained with a crystal violet solution (Beyotime, Shanghai, China) and counted under the microscope (Nikon, Minato, Tokyo, Japan).

### 4.12. Enzyme-Linked Immunosorbent Assay (ELISA)

According to the manufacture protocol, IL-32 protein expressions in CAFs with conditioned LOXL2 were quantified by ELISA (R&D, Minneapolis, MN, USA). The absorbance was detected at 450 nm on a SpectraMax Plus spectrometer. 

### 4.13. RNA-seq and Analyses

According to the Illumina TruSeq RNA Sample Prep Kit protocol, RNA sequencing was performed to detect the expression profiles between siLOXL2 and control samples with Illumina HiSeq 4000 (Illumina, San Diego, CA, USA). Raw counts were aligned to the human genome GRCh38/ hg38 by BWA. R package limma was used for differentially expressed genes (DEGs) identification. 

### 4.14. Statistical Analysis

R software (v 4.1.1) was used to perform all statistical analyses [47]. Shapiro–Wilk test and Levene’s test was used for testing the normality and homogeneity properties, respectively. The distribution of data was firstly visualized by plotting histograms (produced by *hist()* function in R). Then, skewness and kurtosis of data were assessed by *skewness()* and *kurtosis()* functions from the R package moments. Categorical variables were compared through chi-squared test, and Wilcoxon rank-sum test or T test was applied to compare continuous variables. Pearson correlation coefficient was used for correlation analysis. Kaplan–Meier curves with the log-rank test were performed for survival analysis. All experiments were conducted at least three times and data were presented as mean ± standard error (M ± SE). A two-tail *p* value < 0.05 was regarded as significant in statistics.

## 5. Conclusions

Through integrated single-cell and bulk RNA-seq analyses, we identified an iCAF-related signature that stratified BCa patients into two subtypes with distinct molecular features. The signature demonstrated robustness in predicting prognosis and response to immunotherapy/chemotherapy in TCGA-BLCA, GEO-meta, and three ICI-treated cohorts. Subsequent analyses identified the hub gene LOXL2 that predicted poor prognosis, impaired immune reaction, and M2 infiltration. Moreover, LOXL2 was exclusively upregulated in one subpopulation of iCAFs, named “LOXL2+ iCAFs”. Furthermore, in vitro co-culture experiments verified the role of the LOXL2-IL32 axis in regulating bladder cancer cells’ proliferative, migration, and invasive abilities. LOXL2 may serve as a promising target for BCa treatment.

## Data Availability

The original contributions presented in the study are included in the article/Supplementary Material, further inquiries can be directed to the corresponding author.

## Supplementary Materials

The following supporting information can be downloaded at: https://www.mdpi.com/article/10.3390/ijms232415970/s1.

## Abbreviations

iCAFs: inflammatory cancer-associated fibroblasts; BCa: bladder cancer; TME: tumor microenvironment; NMIBC: non-muscle invasive bladder cancer; MIBC: muscle-invasive bladder cancer; TEX: T-cell exhaustion; ssGSEA: single-sample Gene Set Enrichment Analysis; ORA: Over Representation Analysis; PPI: protein–protein interaction; DMEM: Dulbecco’s modified Eagle’s medium; qRT-PCR: quantitative real-time reverse transcription-polymerase chain reaction; RT: room temperature; CM: conditioned medium.
