# Supplementary material for: Integrated Analysis Revealed an Inflammatory Cancer-Associated Fibroblast-Based Subtypes with Promising Implications in Predicting the Prognosis and Immunotherapeutic Response of Bladder Cancer Patients
Source: Int J Mol Sci. 2022 Dec 15;23(24):15970. doi: 10.3390/ijms232415970 (PMC9781727; doi:10.3390/ijms232415970)
Supplement: Supplementary file 1 [file ijms-23-15970-s001.zip › Supplementary Figures.pdf]

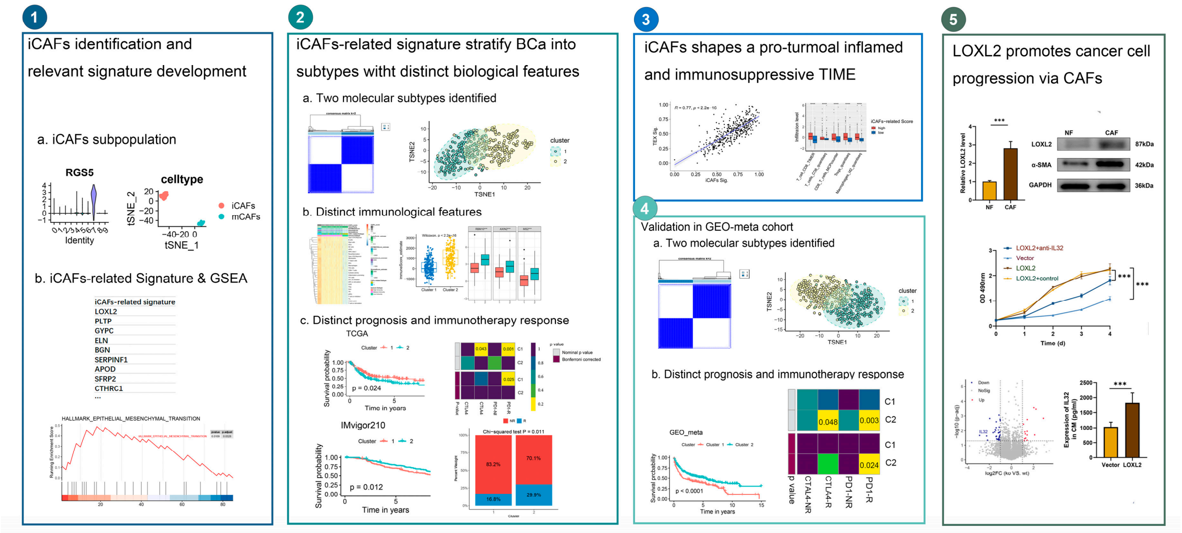

Figure S1. The workflow of the study

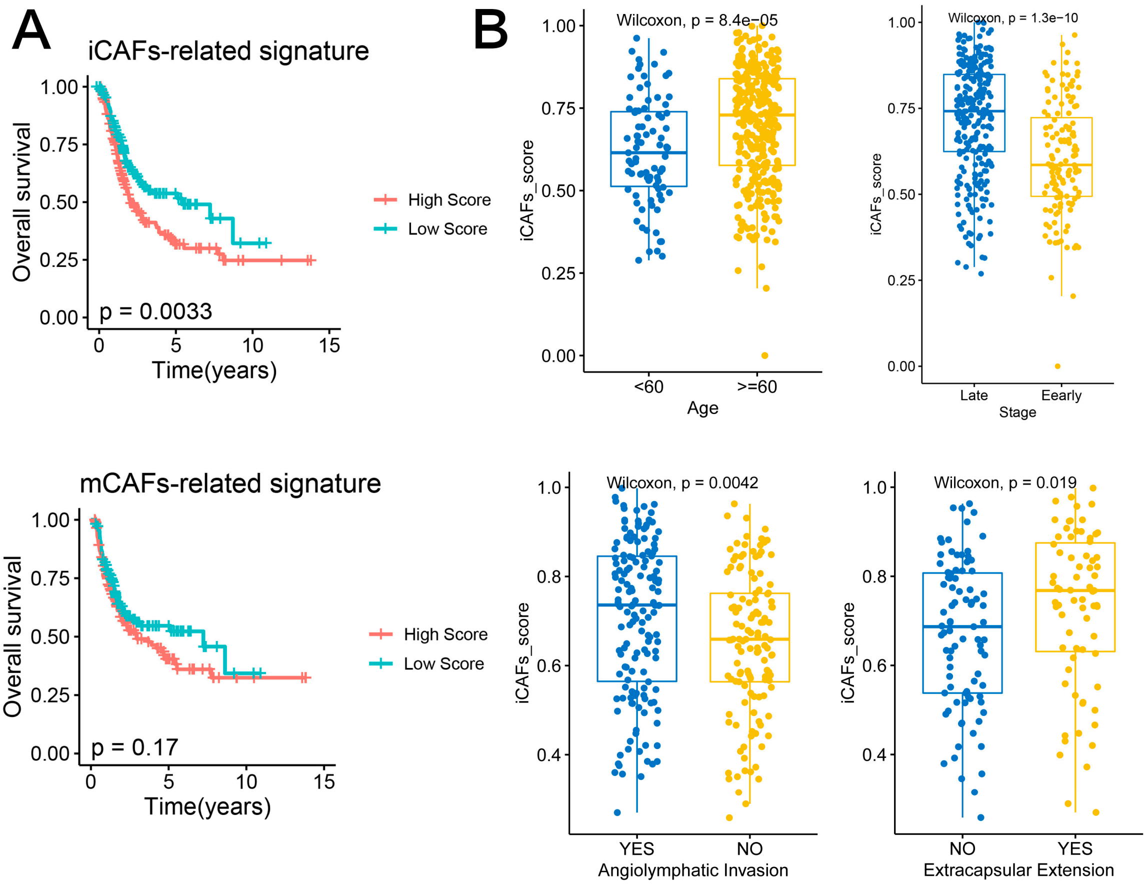

Figure S2. iCAFs subtype was related to poor prognosis and progression of BCa patients: (A) KM curves with log-rank tests demonstrated the survival discrepancy between TCGA-BLCA with high- and low-enrichment scores of iCAFs (up) /mCAFs (bottom). The median value of the score was set as the cutoff. (B) Enrichment scores of iCAFs between clinicopathological factors. Early stage: stage I and II. Late stage: stage III and IV

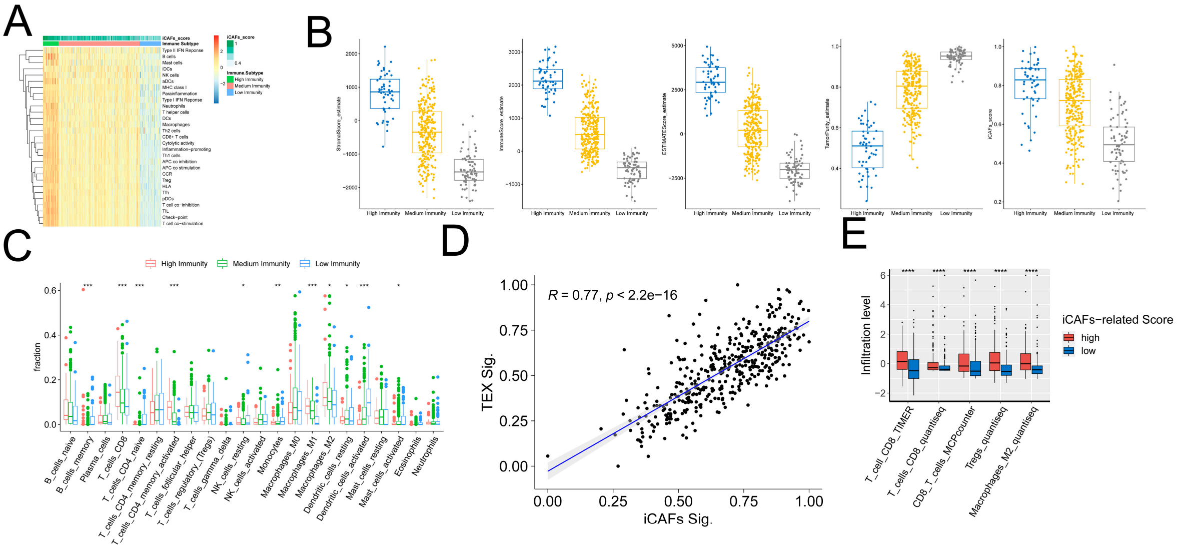

Figure S3. iCAF-related signature dominated an inflamed and immunosuppressive TME of BCa: (A) Three immunity subtypes were identified by unsupervised hierarchical clustering. (B) The distribution of stromal, immune, ESTIMATE score, tumor purity, and iCAF signature across three immunity subtypes. (C) The infiltration abundances of 22 immune cells across three immunity subtypes. (D) The scatter plot demonstrated the Pearson Correlation Coefficient between the iCAF signature and the TEX signature. (E) The infiltration abundances of CD8+ T cells (decoded by TIMER, quanTIseq, and MCPcounter), Tregs (decoded by quanTIseq), and M2 macrophages (decoded by quanTIseq) between high- and low-enrichment score of iCAF signature. The median value of the score was set as the cutoff.

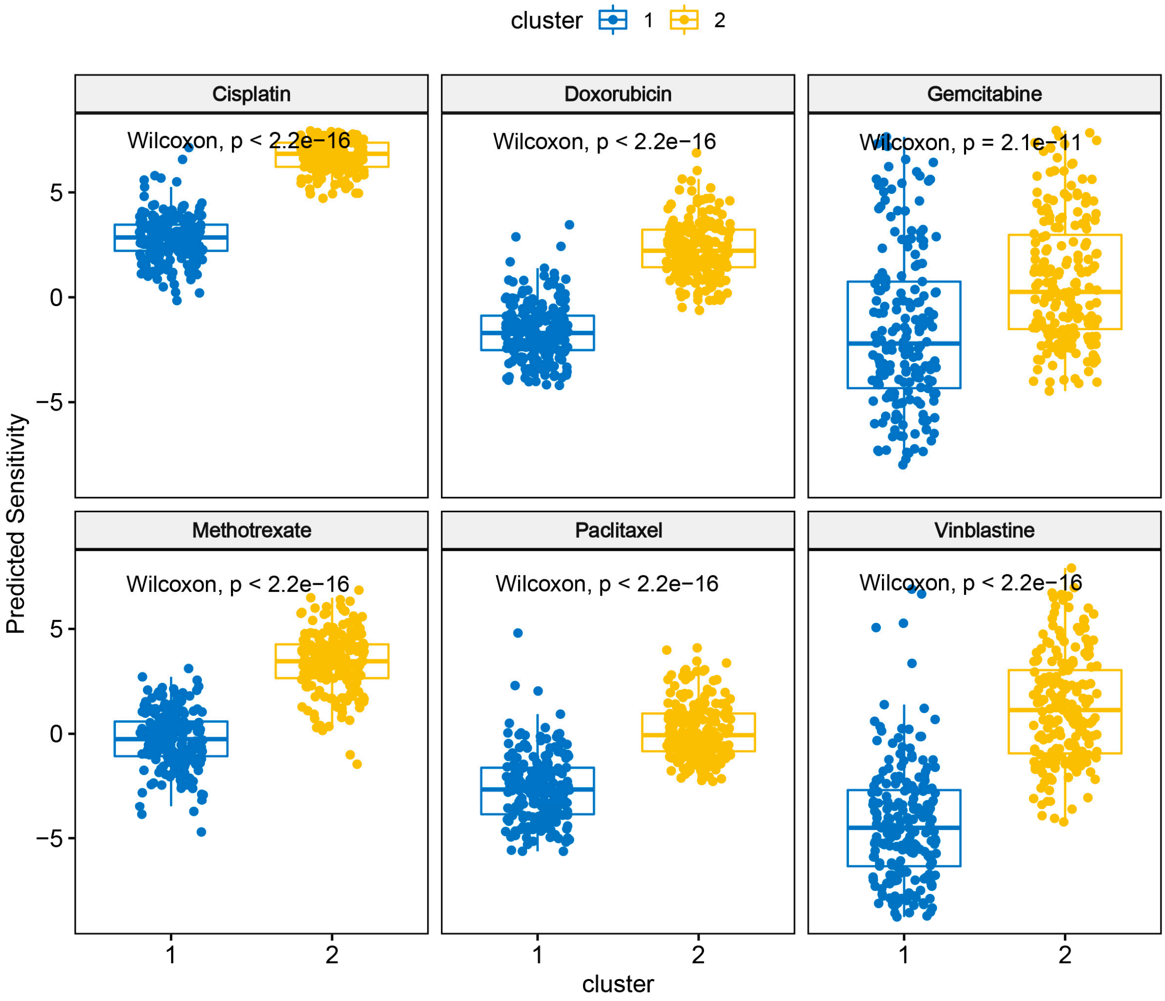

Figure S4. Sensitivity to chemotherapy between two clusters.

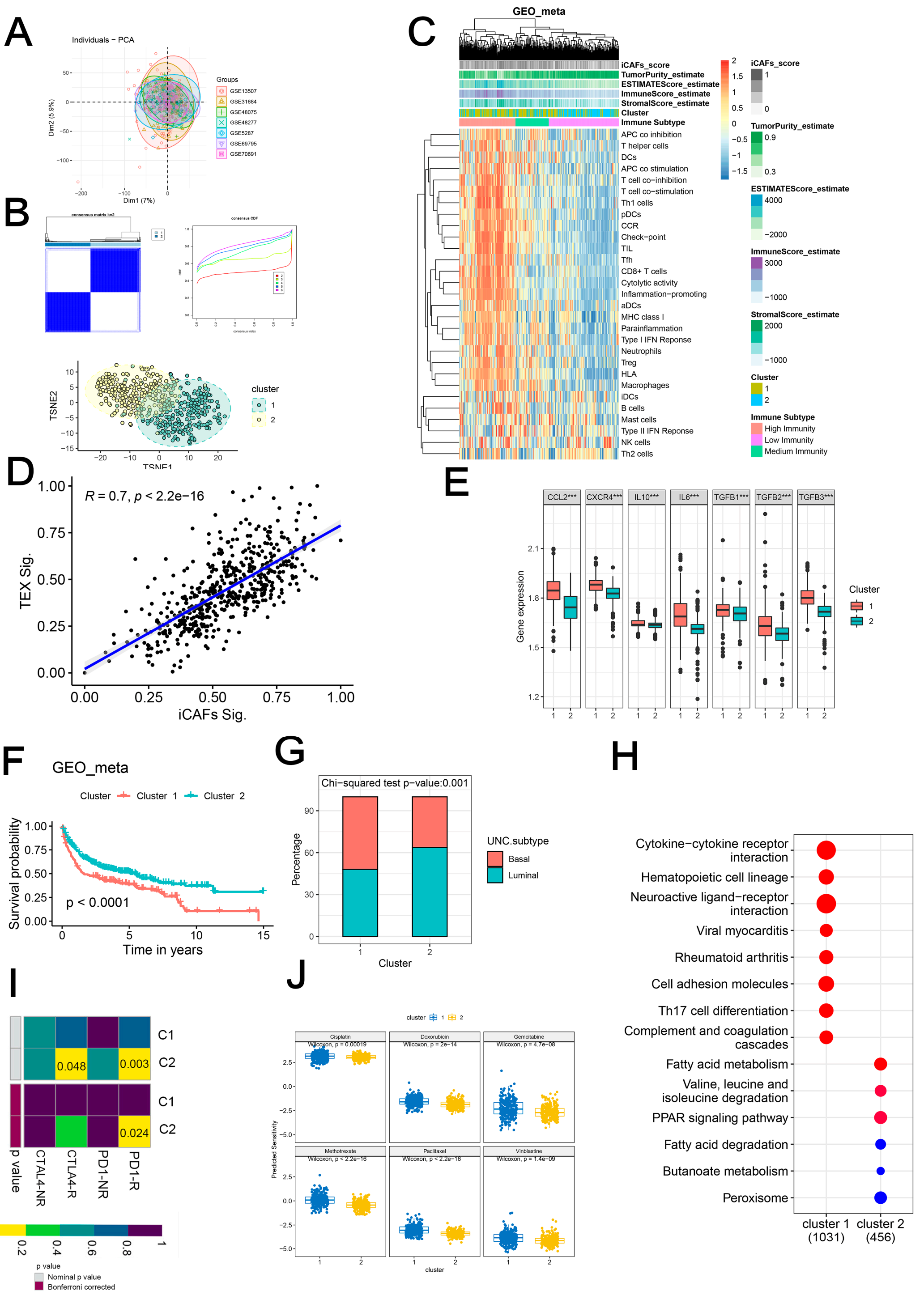

Figure S5. iCAF-related subtypes demonstrated robustness in the external GEO-meta dataset: (A) Batch effects were adjusted in the integrated GEO dataset. (B) The GEO-meta dataset was divided into two molecular subtypes with good discrimination. (C) Heatmap demonstrated three immunity subtypes with diverse iCAF-related subtypes, ESTIMATE score, and iCAF signature score. (D) The correlation between TEX and iCAFs. (E) Cluster 1 was featured with overexpressed M2 macrophage polarization factors. (F) Cluster 1 was related to a worse prognosis in GEO-meta. (G) Consensus UNC molecular subtypes between two clusters. (H) Upregulation pathways between two clusters. (I) Immunotherapy response prediction. (J) Sensitivity to chemotherapy between two clusters.

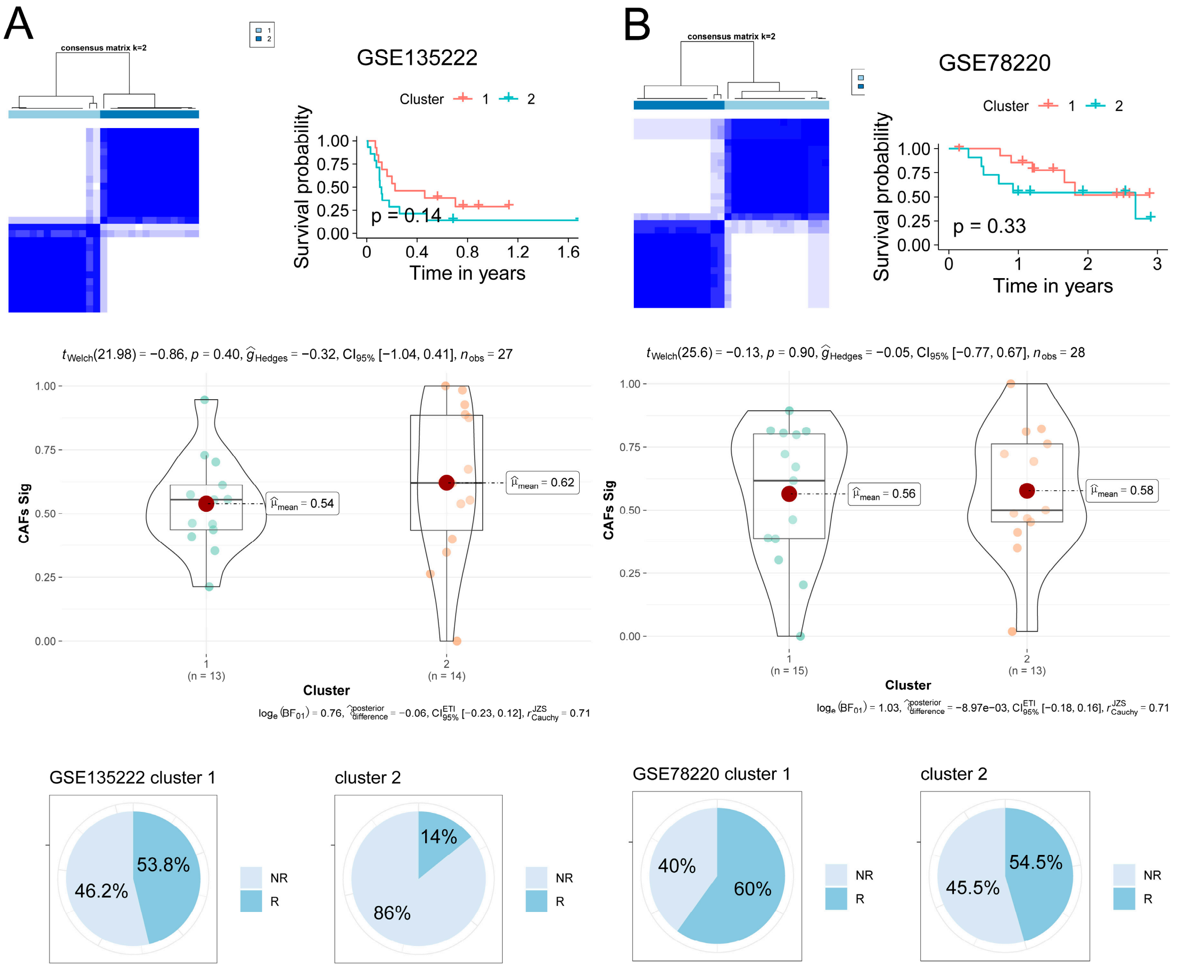

Figure S6. iCAF-related subtypes predicted prognosis and immunotherapeutic response in (A) GSE135222 and (B) GSE78220

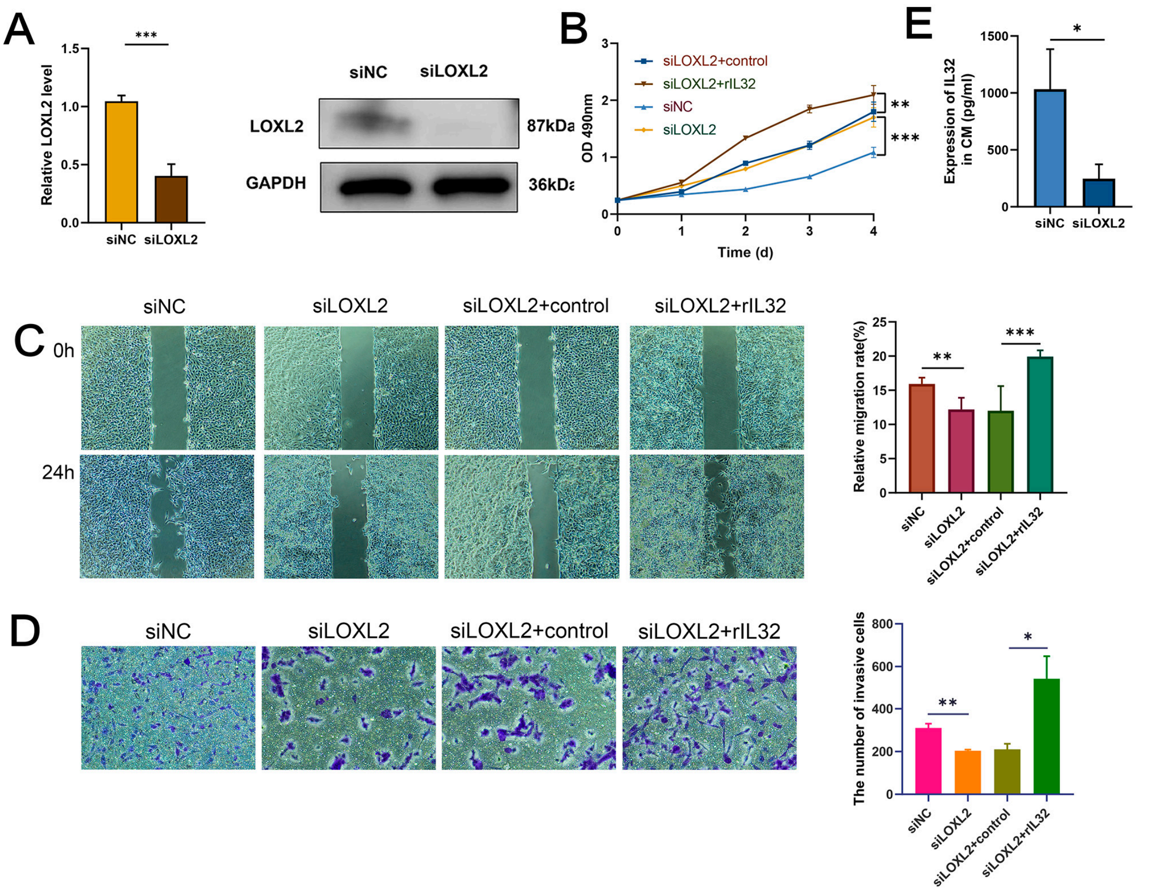

Figure S7. Silencing LOXL2 in CAFs inhibited the proliferation, migration, and metastasis of bladder cancer cells: (A) qRT-PCR and WB verified the efficacy of silencing. (B) CCK-8 assay demonstrated the proliferation effect of LOXL2 in CAFs together with or without rIL32. (C, D) T24 cells were treated the same as in (B), and the wound-healing assay (C) and Transwell assay (D) were carried out to evaluate the migrative and invasive potentials of tumor cells. (E) ELISA demonstrated the IL32 protein expressions in the culture supernatant of CAFs with conditioned LOXL2.
