# Supplementary material for: Integrated Analysis Revealed an Inflammatory Cancer-Associated Fibroblast-Based Subtypes with Promising Implications in Predicting the Prognosis and Immunotherapeutic Response of Bladder Cancer Patients
Source: Int J Mol Sci. 2022 Dec 15;23(24):15970. doi: 10.3390/ijms232415970 (PMC9781727; doi:10.3390/ijms232415970)
Supplement: Supplementary file 1 [file ijms-23-15970-s001.zip › Table S1.pdf]

Table S1. Gene list of iCAFs-related signature

CFD	DCN	MGP	IGFBP7	HMGA1	CD55	HSPD1	TCF4	COMP
PLTP	LUM	VIM	PCOLCE	FGF7	KRT14	LSR	HTRA1	CAV1
GYPC	COL1A1	TMEM45A	COL5A2	CSTB	COL5A1	KRT17	THY1	HCCAT3
ELN	RARRES2	TACSTD2	MXRA8	CLEC2B	PTRF	TYMP	CITED4	ZFP36
BGN	COL1A2	ISLR	RARRES3	EMILIN1	CD99	MTRNR2L2	TMEM119	PRRX1
SERPINF1	COL3A1	DPT	RHOF	FTL	CCND1	LY6E	IGFBP6	NTM
APOD	C1S	ADIRF	IFITM2	DBI	FKBP1A	CTSB	CKS2	ASPN
SFRP2	C1R	TPM2	CXCL2	CLDN3	CDH11	PALLD	HMOX1	SULF1
CTHRC1	MMP2	CDKN2A	SFN	PPARG	HSPB1	IDI1	APOBEC3B	TGFBI
HSPA1A	COL6A3	PSCA	AGPAT2	HLA-B	GPNMB	TUBA1A	S100A4	FIBIN
CXCL14	NUPR1	CTSK	NNMT	UPK1A	UCP2	MAGEA3	KLF6	UPK3A
KRT7	COL6A2	AREG	MFAP4	VCAN	IGFBP3	MT1E	PLAT	LYPD3
SERPING1	AEBP1	SPINK1	C3	PRKCDBP	TAGLN	GJB2	C11orf96	CTGF
SRPX	IGF1	DKK1	EDN1	GEM	NREP	GADD45B	TIMP3	PRSS22
LTBP4	SPARC	A2M	SPON2	CRISPLD2	CCDC80	TPM1	CCL15	AC015849.16
NBL1	TMEM176B	MAP1LC3A	HSPB8	FXYD3	MMP14	MT2A	KRT20	PTGDS
LSP1	LOXL2	HN1	RCN3	IGF2	SFRP4	POSTN	HIST1H2BK	PLAU
CLDN4	KRT19	CCL2	FSTL1	COL8A1	FBLN1	TINAGL1	OLFML3	KIAA0101
COL6A1	IGFBP5	IFITM1	UPK2	TMEM147	MAL	GATA3	TGFB1I1	CLIC3
MMP23B	CALD1	ELF3	PTMS	TNFRSF12A	MAL2	CFH	HMGB3	RBP1
TNFAIP6	PLA2G2A	CRYAB	IDH1	TOMM40	FHL2	SET	FOS	GUCA2A
SELM	GAS6	LGALS3BP	SEPP1	AXL	PDLIM3	PDGFRB	FILIP1L	HIST1H1C
C7	TFPI	CLU	SOD2	ID1	IRF1	MMP11	PCNA	
PLAC9	PRNP	IGFBP4	EMP3	WASH4P	RGCC	MARCKS	HMGB2	
CLEC11A	CYGB	P4HA2	NID2	GPX1	GSN	SDC2	TUBA1B	
IFITM3	LHFP	COX7A1	UPK3B	ID2	THBS2	INHBA	ARHGAP29	
KRT8	S100P	RGS16	CYBA	EGR1	ADM	NQO1	PODN	
SPINT2	SPARCL1	ECM1	MYL9	ARL6IP1	C20orf24	CKS1B	GJA1	
IFI16	CXCL12	SERPINE2	IL32	TMEM176A	AMIGO2	MYADM	SPTSSB	
C19orf33	MEG3	PERP	SERPINH1	GDPD3	CD248	PKIG	IER3	
TIMP1	UPK1B	CST6	EEF2	MFAP2	PNRC1	FAP	MYCL	
KRT18	HSD17B2	CRCT1	HLA-A	PDPN	SAT1	FBLN2	S100A14	
